# Cu-Doped ZnO Thin Films Deposited by a Sol-Gel Process Using Two Copper Precursors: Gas-Sensing Performance in a Propane Atmosphere

**DOI:** 10.3390/ma9020087

**Published:** 2016-01-29

**Authors:** Heberto Gómez-Pozos, Emma Julia Luna Arredondo, Arturo Maldonado Álvarez, Rajesh Biswal, Yuriy Kudriavtsev, Jaime Vega Pérez, Yenny Lucero Casallas-Moreno, María de la Luz Olvera Amador

**Affiliations:** 1 Área académica de Computación y Electrónica, Instituto de Ciencias Básicas e Ingeniería, Universidad Autónoma del Estado de Hidalgo, Hidalgo 56092, Mexico; 2 Departamento de Ingeniería Eléctrica, Centro de Investigación y de Estudios Avanzados del Instituto Politécnico Nacional, México D.F. 14740, Mexico; ejlunaa@cinvestav.mx (E.J.L.A.); amaldo@cinvestav.mx (A.M.A.); yuriyk@cinvestav.mx (Y.K.); molvera@cinvestav.mx (M.L.O.A.); 3 Instituto de Energías Renovables, Universidad Nacional Autónoma de México, Morelos 62580, Mexico; rroshan@ier.unam.mx; 4 Escuela Superior de Ingeniería Mecánica y Eléctrica, Unidad Ticoman del Instituto Politécnico Nacional, México D.F. 07340, Mexico; jvegap@ipn.mx; 5 Departamento de Física, Centro de Investigación y de Estudios Avanzados del Instituto Politécnico Nacional, México D.F. 14740, Mexico; ycasallas@fis.cinvestav.mx

**Keywords:** copper dopant, gas sensor, sol-gel

## Abstract

A study on the propane gas-sensing properties of Cu-doped ZnO thin films is presented in this work. The films were deposited on glass substrates by sol-gel and dip coating methods, using zinc acetate as a zinc precursor, copper acetate and copper chloride as precursors for doping. For higher sensitivity values, two film thickness values are controlled by the six and eight dippings, whereas for doping, three dippings were used, irrespective of the Cu precursor. The film structure was analyzed by X-ray diffractometry, and the analysis of the surface morphology and film composition was made through scanning electron microscopy (SEM) and secondary ion mass spectroscopy (SIMS), respectively. The sensing properties of Cu-doped ZnO thin films were then characterized in a propane atmosphere, C_3_H_8_, at different concentration levels and different operation temperatures of 100, 200 and 300 °C. Cu-doped ZnO films doped with copper chloride presented the highest sensitivity of approximately 6 × 10^4^, confirming a strong dependence on the dopant precursor type. The results obtained in this work show that the use of Cu as a dopant in ZnO films processed by sol-gel produces excellent catalysts for sensing C_3_H_8_ gas.

## 1. Introduction

ZnO is a II–VI compound semiconductor with a wide direct band gap of 3.4 eV at room temperature. Zinc oxide thin films (ZnO) have been studied with an emphasis on applications as transparent conductive oxide (TCO) coatings for solar cells, light-emitting diodes, bipolar junction transistors, photo-detectors and gas sensors [1,2,3,4,5]. Undoped ZnO is non-stoichiometric, irrespective of the deposition technique used [6]. The material is characteristically an n-type semiconductor due to the non-stoichiometry associated with oxygen vacancy and/or an excess of Zn metal acting as donor states providing conduction electrons to the host lattice. In addition, the superficial electrical resistance of ZnO thin films is influenced by the adsorption of oxygen from the air onto the film surface. It is worth noting that the oxygen adsorption is also affected by the grain size, effective surface area, dopant concentration, pore volume, number of oxygen vacancies, localized donor and acceptor states and other lattice defects formed during the synthesis. When one oxygen ion is adsorbed onto the surface of the ZnO thin film, it traps one or two conduction electrons to produce negatively-charged species ($O_{2}$ or $O_{2}^{-}$, depending on the operating temperature) [7]. The net effect is an increase in the surface electrical resistance of the ZnO thin film. Now, in the presence of reducing gases, like hydrocarbons, at a certain operating temperature, the trapped electrons are released to the conduction band, resulting in a decrease in the surface electrical resistance. The overall reaction of hydrocarbon molecules with adsorbed oxygen can be expressed as follows [8]:

(1) $$C_{n}H_{2n+2}+2{O^{-}}_{\left(\text{ads}\right)}\to /\text{Heat}/\to H_{2}O+C_{n}H_{2n}:O+e^{-}$$

(2) $$C_{n}H_{2n}:O+{O^{-}}_{\left(\text{ads}\right)}\to {\text{CO}}_{2}+H_{2}O+e^{-}$$

where C*_n_*H_2*n*+2_ represents methane (CH_4_), propane (C_3_H_8_) or butane (C_4_H_10_) and C*_n_*H_2*n*_:O represents partially-oxidized intermediate compounds on the ZnO surface.

The adsorption of gas molecules that underlies the gas-sensing properties of undoped ZnO thin films is usually modified using a catalyst. Different elements have been used for this purpose, including Sn, Al, Fe, Pd and Y [9,10,11,12,13]. Many works have been published on the use of Cu for sensing different gases [14,15,16,17]. It is assumed that Cu^1+^ ions substitute for the Zn^2+^ in the ZnO lattice, reducing the electron concentration, and hence, a higher electrical resistivity is observed [18,19,20,21,22]. Cu-doped ZnO thin films have been prepared by several deposition techniques, such as pulsed-laser deposition, magnetron sputtering, spray pyrolysis and the sol-gel process [23,24,25,26]. The sol-gel technique offers a simple, low-cost and large-area thin-film coating method as an alternative to vacuum deposition techniques.

For Cu-doped ZnO, the stability of the Coulomb forces of the interactions between the acceptor defects (${\text{Cu}}_{\text{Zn}}^{+}$) and intrinsic ZnO donors, namely, zinc interstitials or oxygen vacancies (Zn*_i_* or V_O_) can occur by the capture of an electron from the lattice. A model of an associate donor–acceptor for Cu_Zn_ was proposed by West *et al.* [27], where:

(3) $${\left[{\text{Cu}}_{\text{Zn}}^{+}\left(3d^{10}\right)\right]}^{-}+{\text{Zn}}_{i}^{+}\left(4s^{1}\right)\to {\left\{\left[\text{Cu}\right]{\text{Zn}}_{i}^{+}\left(4s^{1}\right)\right\}}^{0}$$

or, in the Kröger–Vink notation [28],

(4) $${\text{Cu}}_{\text{Zn}}^{*}+{\text{Zn}}_{i}^{*}\to {\left[{\text{Cu}}_{\text{Zn}}-{\text{Zn}}_{i}\right]}^{x}$$

The created complex defects (Equation 3) in Cu-doped ZnO can increase the surface potential barrier for electrons in the conduction band. In general, the amount of adsorbed oxygen species on the surface would depend on the Cu atoms in the ZnO, which in turn would oxidize the exposed gas, as shown in Equations 1 and 2. Consequently, as the Cu concentration on the ZnO surface increases, it leads to a higher sensibility due to the increase in the amount of adsorbed oxygen on the film surface [29].

On the other hand, as the hydrocarbon molecules approach the ZnO surface, reorientation of the electric charge takes place to form induced dipoles. As a result, such hydrocarbon molecules are attracted towards the active electrical sites, leading to this adsorption [30].

The effect of the absorption of induced dipoles on the surface of the grains is to create an electric field and thereby alter the height of the surface potential barrier. If the electric field produced by such dipoles is opposed to the surface electric field, then the potential barrier decreases. Thereby, the transport of the charge carriers between grains increases, which in turn increases the surface electric conductivity (*G*). On the other hand, if the electric field produced by these dipoles points to the direction of the electric field on the surface, then the potential barrier increases, hindering the transport of charge between grains, so that the surface electric conductivity decreases [31,32].

Therefore, in the case of the adsorption by induced dipoles, the gas molecules are adsorbed completely. Although this type of adsorption does not cause an increase in the concentration of charge carriers, it affects the potential barrier present on the surface and, thereby, the transport of these charge carriers.

In the case of adsorption by dissociation, described in Equations (1) and (2), the hydrocarbon molecules can be adsorbed and decomposed by reacting with the oxygen desorbed from the surface. In this process, there is an increase in the charge carrier concentration and a decrease in the height of the surface potential barrier.

In related works, the adsorption mechanisms of hydrocarbon gases on a semiconductor surface are explained in terms of Equations (1) and (2). However, the analysis of samples by the highly sensitive and powerful secondary ion mass spectroscopy (SIMS) technique shows that complex compounds derived from secondary chemical reactions were also formed when propane gas interacts with the ZnO surface. 

The surface morphology also plays an important role in the adsorption of gas, because as the effective surface area or contact area of ZnO, suitable for gas adsorption, increases, so does the sensitivity. The adsorption energy at the surface of the grains or the energy required to capture molecules is a function of the ratio of empty volume/grain volume (porosity). If this ratio is small, the adsorption energies at the grain surface interfere with each other, causing the adsorption energy to rise. In this case, the increase in the adsorption energy at the grain surface causes an increase in the ability to capture more molecules [33,34,35]. Therefore, it is anticipated that tailoring both the effective surface area and the ratio of empty volume/grain volume can lead to a substantial improvement in sensitivity.

If both the value of the ratio of empty volume/grain volume and the grain size are small, the sensitivity improves [36,37]. The reduction of the grain size to nanometers or to a scale comparable to the thickness of the charge depletion layer leads to a dramatic improvement in the sensitivity [38,39]. It is found that also the crystal structure of the grains affects the absorption of gases [40]. Similarly, it is found that certain preferential orientations help to improve the gas adsorption [41].

In this work, the sensing properties of Cu-doped ZnO thin films deposited by the sol-gel technique in a propane atmosphere were analyzed. The criteria for sensing propane (C_3_H_8_) are because it occupies one of the main positions among all combustible and explosive gases and also due to its high demand for domestic tasks [42]. Two different Cu precursors were used to prepare the starting solutions. The film thickness effect on the film’s morphology and sensing properties was studied. Additionally, the structural and compositional properties of Cu-doped ZnO post-sensed films and a comparison of the CO gas-sensing performances of Cu-doped ZnO films were analyzed to obtain more information about the sensing mechanisms.

## 2. Experimental Procedure

### 2.1. Deposition Conditions

Undoped ZnO thin solid films were deposited onto soda lime glass substrates from a 0.6 M starting solution prepared from zinc acetate dehydrate (Zn(CH_3_CO_2_)_2_·2H_2_O, 99.5% purity, Merck, S.A. de C.V., Naulcalpan de Juarez, Edo. de México, México) dissolved in a mixture of 2-methoxyethanol (2-MOE) (CH_3_OCH_2_CH_2_OH, 98% purity, Sigma-Aldrich Quimica, S.L., Toluca, Edo. de México, México) and monoethanolamine (MEA) (NH_2_CH_2_CH_2_OH, Sigma-Aldrich, St. Luis, MO, USA, 98% purity) at room temperature by the sol-gel and dip-coating processes. The MEA is used for improving the zinc precursor solubility and to stabilize the starting solutions. The solutions were prepared under constant magnetic stirring at room temperature for 1 h, until the solution pH reached approximately 8. These undoped ZnO films were used as a reference in the structural characterizations and sensing measurements. For depositing Cu-doped ZnO films, two different copper precursors, namely copper chloride [CuCl_2_] and copper acetate [CuC_2_H_3_O_2_], both dissolved in 2-MOE, were used.

The dip-coating process consists of repeated dipping and drying steps: 6 and 8 dips were used for undoped samples for observing the thickness difference and 3 dips in the copper solutions for depositing Cu-doped ZnO films. An annealing process in air at 200 °C was carried out for 10 min after every immersion to dry and remove the residual solvents. Finally, to improve the film homogeneity, a second annealing process was performed at 450 °C in air for 1 h.

### 2.2. Characterization Conditions

The structural characteristics of the deposited ZnO films were investigated by X-ray diffraction (XRD) with a Siemens-Kristalloflex diffractometer using Cu-Kα radiation, under the θ–2θ method. The 2θ scanning range was from 20° to 70°. The average crystallite size for the Cu-doped ZnO films was calculated from the (002) line, using Scherrer’s equation [43]. The concentration profiles of the species in films were obtained from SIMS measurements, carried out by a CAMECA IMS-6F Ion Microprobe CAMECA (Instruments, Inc., Madison, WI, USA) equipped with a cesium ion gun and duoplasmatron ion sources.

To determine the concentrations of atoms $C_{M}$, the secondary ion intensities $I_{s}^{M}$ were monitored. The relationship between the intensity of a secondary ion and the concentration of atoms is given by:

(5) $$I_{s}^{M}=I_{P}Y\alpha_{M}^{\pm }\beta_{M}C_{M}$$

where *I*_p_ is the primary ion beam current produced by *M* atoms, *Y* is the atomic erosion yield, $\alpha_{M}^{\pm }$ is the ionisation probability, $\beta_{M}$ is an instrumental transmission factor and *C_M_* is the concentration of the atoms under investigation [44]. The values of the constants $I_{P},\;Y,{\;\alpha }_{M}^{\pm }{\text{ and }\beta }_{M}$ can be calculated for a sample with *M* chemical atoms, and *C_M_* is its known concentration or its reference known pattern. Similarly, for a sample with *M* atoms and an unknown *C_M_* concentration or a lack of a reference pattern, *C_M_* can be calculated as long as the constants $I_{P},Y,{\;\alpha }_{M}^{\pm }{\text{ and }\beta }_{M}$ are known.

The films thickness of the Cu-doped ZnO thin films was measured by a KLA Tencor profilometer, Model P15, (LabX, Midland, ON, Canada). The surface morphology of the thin films was observed using a field emission scanning electron microscopy (FESEM, LabX), Carl Zeiss Auriga 39-16 equipment, with an accelerating voltage of 20 keV.

The sensing properties, reported as “*S*”, were calculated from the normalized conductance change, given by the difference between the electrical conductivity measured in the presence of propane gas (*G*_G_) and the electrical conductivity measured in air (*G*_O_) [45]:

(6) $$S=\frac{G_{G}-G_{O}}{G_{O}}$$

The electrical conductance was measured using two rigid tungsten probes, separated by 1 cm. High-purity silver paint was used to manufacture the ohmic contacts on the sample surface.

## 3. Results and Discussion 

### 3.1. Crystal Structure of the Films

**Figure 1:**
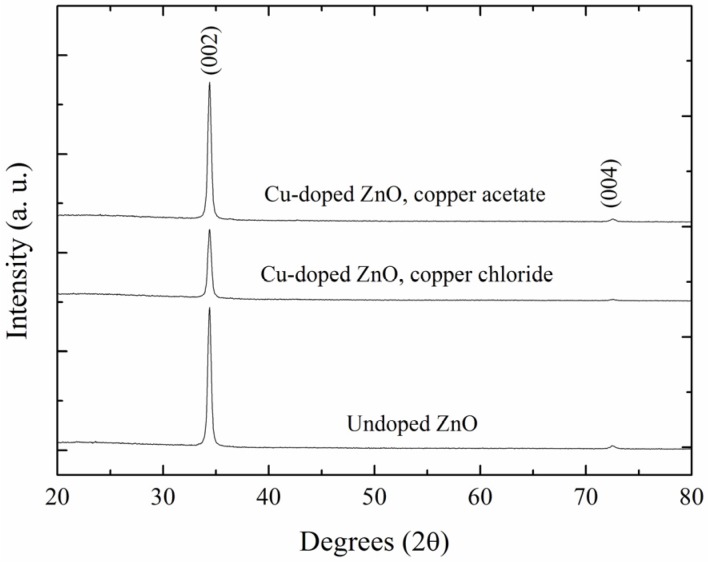
XRD patterns of undoped ZnO and Cu-doped ZnO films.

The X-ray diffraction spectra of undoped ZnO and Cu-doped ZnO films prepared from two different Cu precursors (copper chloride or copper acetate) by deposition with eight immersions are shown in Figure 1. All spectra present a main diffraction peak at approximately 34.4° and another with very low intensity at 72.7°, corresponding to the (002) and (004) planes, respectively, of the ZnO hexagonal wurtzite structure, according to JCPDS Card No. 36-1451 [46]. The strong preferential orientation in the (002) plane indicates that thin films exhibit good crystallinity, which corresponds to the most stable phase of ZnO [47]. Additionally, it is evident that no extra phases corresponding to either copper or copper oxides were detected from this technique. 

The average crystallite size, *D*, and the lattice constant, c, of the undoped and Cu-doped ZnO films were estimated from the X-ray spectra. *D* was deduced from the (002) peak using the following Scherrer’s formula:

(7) $$D=\frac{0.89\lambda }{\beta \text{cos}\theta }$$

where λ is the wavelength of Cu-Kα radiation (λ = 1.5405 Å), θ the diffraction angle and β the full width of the diffraction line measured at half of its maximum intensity, in radians.

The lattice constant c was estimated from the XRD data using the following equations, given for hexagonal structures:

(8) $$d_{002}=\frac{\lambda }{2{\text{sin}\theta }_{002}}$$

(9) $$d_{hkl}=\frac{1}{\sqrt{\left[4\left(h^{2}+hk+k^{2}\right)/3a^{2}\right]+l^{2}/c^{2}}}$$

where *d*_002_ is the interplanar distance along the (002) crystallographic direction, obtained from Bragg’s law, and θ_002_ is the diffraction angle of the (002) peak [48].

The lattice constant c reported for bulk ZnO (5.204 Å [49]) is smaller than that estimated from our thin films, due to the relaxed lattice. As seen in Table 1, the undoped ZnO thin films have the best structural properties, *i.e.*, the crystallite size *D* is the largest, and the values of the lattice constant c and angle of diffraction 2θ are close to the ZnO bulk values.

The X-ray diffraction spectrum of the undoped ZnO films is similar to that of the Cu-doped ZnO films, and the slight difference (Table 1) in the latter’s lattice parameters is probably caused by the Cu atoms occupying different positions in the wurtzite ZnO lattice. The Cu concentration was obtained from SIMS measurements, and the results are presented in Section 3.3.

**Table 1:** Full width half maximum (FWHM or β), angle of diffraction (2θ), lattice constant (c) and average crystallite size (*D*) for undoped ZnO and Cu-doped ZnO thin films prepared with copper chloride and copper acetate.

ID Sample	FWHM (°)	2θ (°)	c (Å)	*D* (Å)
Cu-doped ZnO, copper chloride	0.2048	34.37	5.212	401.4
Cu-doped ZnO, copper acetate	0.2036	34.38	5.210	403.8
Undoped ZnO	0.2032	34.39	5.207	405.5

### 3.2. Morphological Properties

**Figure 2:**
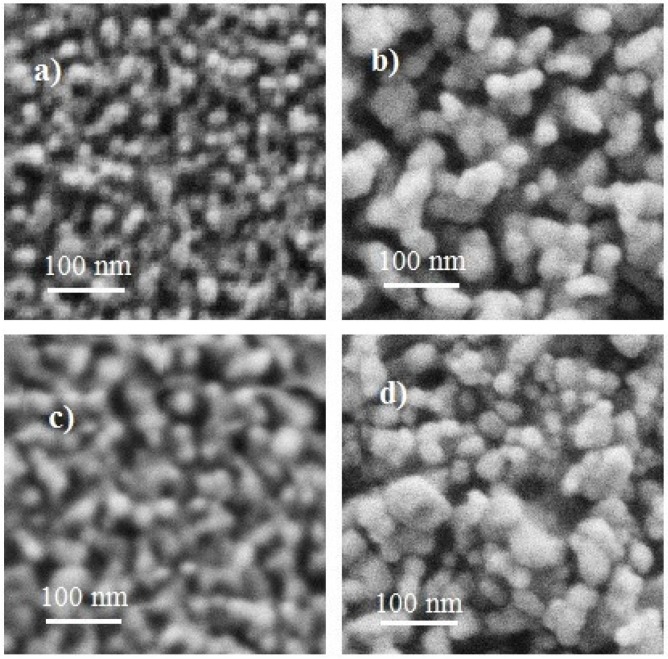
SEM micrographs of Cu-doped ZnO thin films prepared from copper chloride by (**a**) six and (**b**) eight dips and Cu-doped ZnO thin films prepared from copper acetate by (**c**) six and (**d**) eight dips.

Figure 2 shows the surface morphology of the films analyzed using the SEM technique. In general, ZnO films produced by the sol-gel technique exhibit a porous surface with spherical-shaped nano-sized secondary grains [50]. The morphological properties of the semiconductor oxide are influenced by the film thickness, deposition technique, incorporation of dopant and dopant solution, among other factors [51,52]. For example, rough surfaces of Ga-doped ZnO thin films [53] and smooth surfaces of Al-doped ZnO thin films [54] were obtained using the spray pyrolysis technique. In this study, it can be clearly observed that the grain size of the surface increases with the film thickness, and there is a non-uniform grain size distribution on the surface. Changes in the film thickness and doping solution play important roles in the size of the grain. 

In these figures, the effect of the film thickness on the grain size can be clearly seen. As can be seen in Figure 2a, for films deposited from the doping solution of copper chloride by six immersions, the grain size varies between 5 and 10 nm, and for films deposited from the same doping solution by eight immersions, the grain size increases to 30 to 60 nm, as depicted in Figure 2b. Hence, it can be inferred that the grains show an increase in size with the increasing film thickness. Similar behaviors were also observed in films deposited from a doping solution of copper acetate by six and eight immersions, having a grain sizes of 10 to 20 nm and 30 to 60 nm, as seen in Figure 2c,d, respectively. From Figure 2, the surface of the films deposited from copper chloride is slightly more porous. In Section 3.4.1, using digital image processing techniques, root mean square (RMS) surface roughness values and the porosities or ratios of empty volume/grain volume were calculated to analyze the effect on the sensor properties.

### 3.3. Composition Analysis

**Figure 3:**
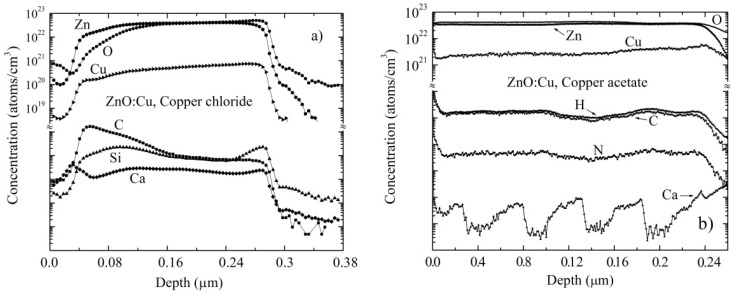
Secondary ion mass spectrometry (SIMS) depth profiles for the six-times dipped Cu-doped ZnO film deposited with (**a**) copper chloride, (**b**) copper acetate.

To determine and distinguish the atoms and molecules in the film, a depth profile of Cu-doped ZnO, obtained from copper chloride (Figure 3a) and copper acetate (Figure 3b), has been plotted from the data obtained from secondary ion mass spectrometry (SIMS). The depth profiles shown in Figure 3 correspond to the six-immersion Cu-doped films, with a thickness of approximately 250 nm. Although the Cu was deposited on the ZnO surface, it is evident that it was effectively incorporated into the films. It can also be seen that the concentration of Cu in the film is less than that of Zn and O, which may be due to the higher binding energy of the O–Zn bond.

The Cu concentration is not constant throughout the film, as it is achieved through inhomogeneous inter-grain spaces or pores in the ZnO film. The distribution of elements on the surface is different for each film. In the case of the ZnO film doped with copper chloride, as shown in Figure 3a, the concentration of the elements is lower in the surface than in the volume, as the measurement was performed immediately after depositing the film. For the case of the ZnO films doped with copper acetate, depicted in Figure 3b, the concentration of elements is higher in the surface than in the volume, which is because the measurement was performed after a long time after the deposition of the film. As a result, the surface-absorbed elements from the atmosphere, such as hydrogen, carbon dioxide and nitrogen, some other elements, such as silicon and calcium, could have diffused from the soda lime glass.

Due to the lack of reference patterns for the atoms of H, C, Si, Ca and Ni, the values of *I*p, *Y*, $\alpha_{M}^{\pm }$ and β*M* were not calculated. Therefore, only the depth profiles are only shown in Figure 3a,b.

The concentration of copper is estimated to be 6.2 × 10^20^ and 2.5 × 10^21^ cm^−3^ for ZnO films doped by copper chloride and copper acetate, respectively. The exact sites occupied by copper atoms within the crystal lattice of the ZnO are unknown, but it is observed that copper acetate is found to be more efficient in incorporating more copper atoms into the crystal lattice of the ZnO.

### 3.4. Sensing Properties

**Figure 4:**
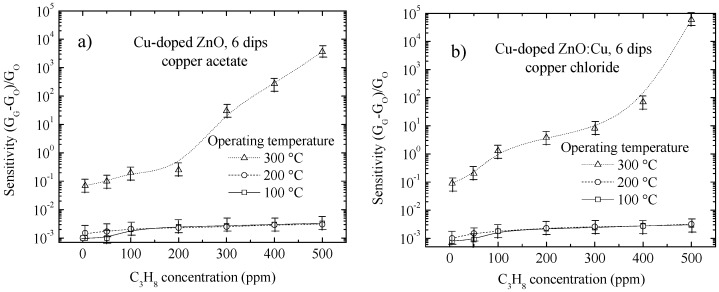
Variation of the sensitivity with propane gas concentrations at different operating temperatures for Cu-doped ZnO films prepared from six dips in (**a**) copper acetate and (**b**) copper chloride.

**Figure 5:**
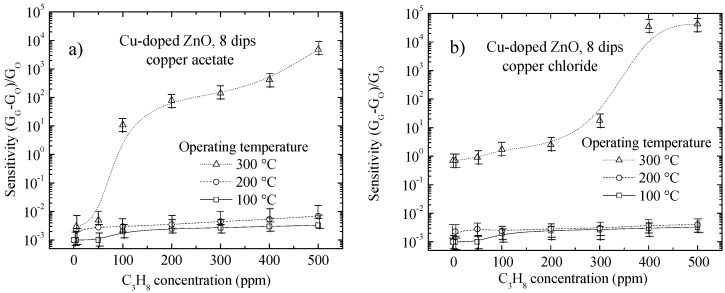
Variation of the sensitivity with the concentrations of propane gas at different operating temperatures for Cu-doped ZnO films prepared from eight immersions in (**a**) copper acetate and (**b**) copper chloride.

As seen in Figure 4 and Figure 5, the sensitivity of Cu-doped ZnO films deposited from the doping precursors of copper acetate (Figure 4a and Figure 5a) and copper chloride (Figure 4b and Figure 5b) prepared with different numbers of dips was analyzed and plotted as a function of the C_3_H_8_ gas concentration ranging between 0 and 500 ppm, at operating temperatures of 100, 200 and 300 °C. To corroborate the reproducibility of the Cu-doped ZnO films, each data point is the mean of the sensor response values for two different samples of the same type.

At room temperature, all samples, both undoped and Cu-doped ZnO films, exhibited very high surface electrical resistances, approximately 600 MΩ for 0 ppm of C_3_H_8_. Nevertheless, a significantly lower electrical resistance, 9 KΩ, was reached when the samples were exposed to 500 ppm of C_3_H_8_ at an operating temperature of 300 °C.

As shown in Figure 4 and Figure 5, the dependence of the sensitivity behavior on the operating temperature and gas concentration was similar for all Cu-doped ZnO films deposited, irrespective of the number of dips or type of doping precursor used. However, there were differences, which are discussed below. It was observed that there is a unique efficient operating temperature with a high sensor response values, which is approximately 300 °C. Therefore, it is necessary to have a high thermal energy available on the surface to promote chemical reactions, which enhance the electrical conductivity of the surface. In the following sections, the effect of each proposed phenomenon is analyzed separately.

#### 3.4.1. Effect of Surface Morphology (Porosity) on Sensing Properties

The effect of the porosity or ratio of empty volume/grain volume on the sensing properties of Cu-doped ZnO thin films is analyzed. It is observed that increasing the number of dips in the solution to deposit the ZnO films increases the thickness of these ZnO films. The results are listed in Table 2.

The gas-sensing performance was found to be strongly dependent on the number of dips (film thickness). This phenomenon could be interpreted in terms of the change in the grain size, average crystallite size, effective surface area and porosity or empty volume/grain volume [31]. The effective surface area or contact area suitable for gas adsorption of the ZnO films increases with the number of dips. Therefore, as the number of dips increases, the grain size and the grain height increases subsequently (Figure 2). Therefore, we can expect that the increase in number of dips will increase the gas sensor sensitivity of the ZnO films. However, for Cu-doped ZnO films deposited with more than six dips, the effective surface area no longer increases, as the sensitivity becomes saturated, which can be seen in Figure 4 and Figure 5.

Due to the unavailability of the grain heights, the effective surface area was not calculated. However, RMS surface roughness values and ratios of empty volume/grain volume were obtained using digital image processing, as shown in Table 3, with an error of ±5% [55]. The height of the tallest grain was considered as unity, and consequently, the heights of the other grains resulted in being fractions of unity, which draws the RMS surface roughness as dimensionless. It can be seen from Table 3 that the roughness increases with respect to the increase in the number of dips. This is due to the increase in grain size. In a similar way, the ratio of empty volume/grain volume decreased as the grain size increased.

**Table 2:** Number of dips and corresponding film thicknesses.

Film	Film Thickness for 6 Dips (nm)	Film Thickness for 8 Dips (nm)
Cu-doped ZnO (copper chloride)	300	350
Cu-doped ZnO (copper acetate)	242	335

From Table 3, it can be observed that the Cu-doped ZnO films prepared from copper acetate have slightly less empty volume or are less porous (considering an error of ±5%) compared to films prepared from copper chloride. Hence, as discussed in the Introduction*,* it may be assumed that the sensitivity of Cu-doped ZnO films prepared from copper acetate should be slightly better at concentrations below 300 ppm C_3_H_8_ (Figure 4 and Figure 5). Additionally, having less empty volume or more grains contributes to a larger effective surface area, and hence, the films prepared from copper acetate must have better sensitivities at high C_3_H_8_ concentrations (above 300 ppm). However, the values of the sensitivities are different for each film, as shown in Figure 4 and Figure 5.

**Table 3:** RMS surface roughness and ratio of empty volume/grain volume of Cu-doped ZnO thin films prepared from copper chloride and copper acetate by 6 and 8 dips.

Morphological Parameters for Different Number Dips	Cu-Doped ZnO (Copper Acetate)		Cu-Doped ZnO (Copper Chloride)	
Number of dips	6	8	6	8
RMS surface roughness	54.32	60.94	48.80	54.50
Empty volume/grain volume (%)	54.62	46.88	58.06	48.98

The behavior of the sensitivity can be explained by the value of the effective surface area, but because of the lack of data for the grain heights, it was not possible to report it. An increase in the grain height causes an increase in the overall grain surface area, which leads to a higher adsorption of gas molecules, thus increasing the sensitivity.

Therefore, it was thought that the Cu-doped ZnO films prepared with copper chloride are composed of taller grains than the Cu-doped ZnO films prepared with copper acetate. Because the Cu-doped ZnO films prepared with copper chloride have the largest film thickness, as can be seen in Table 3, they would have a larger effective surface area, which would explain their high sensitivity, at concentrations of C_3_H_8_ higher than 300 ppm, as shown in Figure 4 and Figure 5.

#### 3.4.2. Adsorption of Propane Gas over the Cu-Doped ZnO Surface

In the Introduction section, two mechanisms of gas adsorption on the Cu-doped ZnO surface were mentioned. In general, the sensitivity is increased starting from an operating temperature of 300 °C and over the full range of gas concentrations. We propose that the dominant mechanism of the adsorption of the propane gas is attributed to the dissociation of molecules. The dissociated C_3_H_8_ molecules react with oxygen species on the grain surface, causing the release of electrons into the conduction band of the ZnO, which in turn decreases the surface potential barrier, leading to an increase in the electrical conductivity. The results of sensing performed on the Cu-doped ZnO films are presented in Figure 4 and Figure 5.

**Figure 6:**
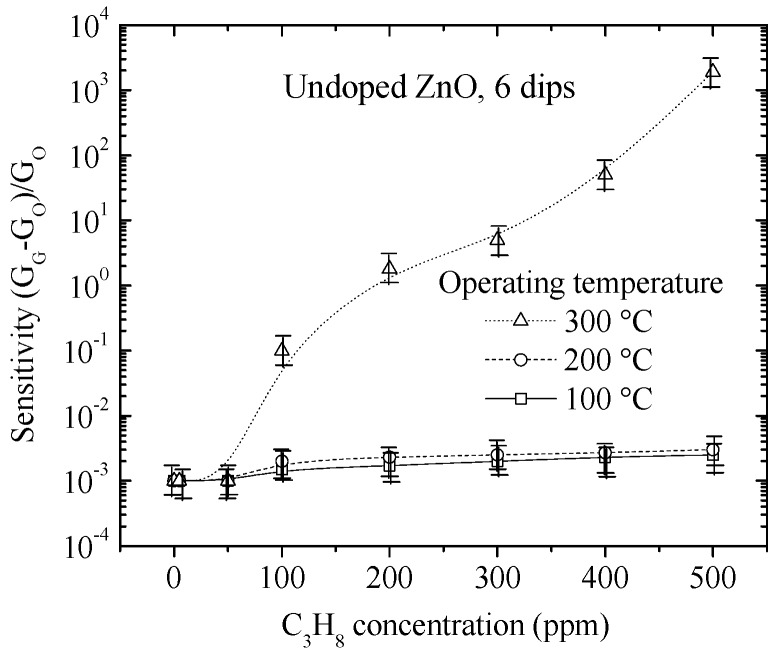
Sensitivity of an undoped ZnO film prepared with six dips as a function of the C_3_H_8_ concentration at different operating temperatures.

An undoped ZnO film is deposited from six dips in zinc acetate solution by the sol-gel process, which is used as a reference and characterized in the same way, at three operating temperatures of 100, 200 and 300 °C, as shown in Figure 6. The sensitivity behavior of the undoped ZnO films was similar to that of the Cu-doped ZnO films, except that the undoped ZnO film presented sensitivity values lower than those of the Cu-doped ZnO films. The maximum sensitivity obtained was approximately 1900, measured at the operating temperature of 300 °C and at the highest propane concentration of 500 ppm. The catalytic effect of the Cu on the sensing properties of the ZnO thin films is therefore self-explanatory. Gas molecules adsorbed onto the Cu-doped ZnO surface react preferentially on the Cu sites, as explained in the Introduction section (refer to Equations (3) and (4)). 

#### 3.4.3. Structural and Compositional Analysis of Cu-Doped ZnO Post-Sensed Films Obtained from Copper Chloride and CO Gas Sensitivity

**Figure 7:**
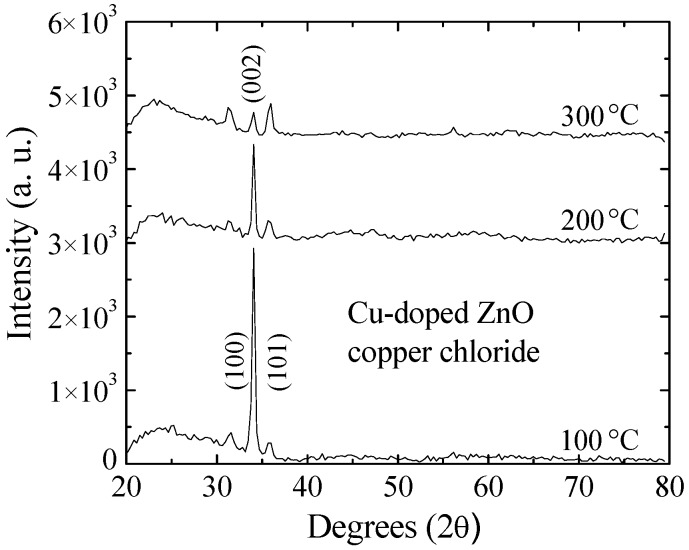
XRD patterns of Cu-doped ZnO post-sensed films prepared from six dips in copper chloride at different operating temperatures.

To obtain more information about the sensing mechanisms, X-rays diffraction patterns and SIMS depth profiles of Cu-doped ZnO post-sensed films were obtained. Figure 7 presents the X-ray diffraction patterns of Cu-doped ZnO films prepared from six dips of zinc acetate and three dips of copper chloride. These Cu-doped ZnO films were used to sense propane gas at three different operating temperatures, *i.e.*, 100, 200 and 300 °C, and under the same gas concentration of 500 ppm. From the results, it is found that as the operating temperature increases, the crystallinity of the ZnO films worsens. Therefore, it can be inferred that the operating temperature favors the chemical reaction between the propane gas and the Cu-doped ZnO surface, which proves that the adsorption of the gas molecules is through chemisorption, as the crystal structure of ZnO has changed [56]. Although it is not presented in this paper, it is well documented that after the first test of the sensing, the sensing properties of the Cu-doped ZnO films begin to deteriorate.

**Figure 8:**
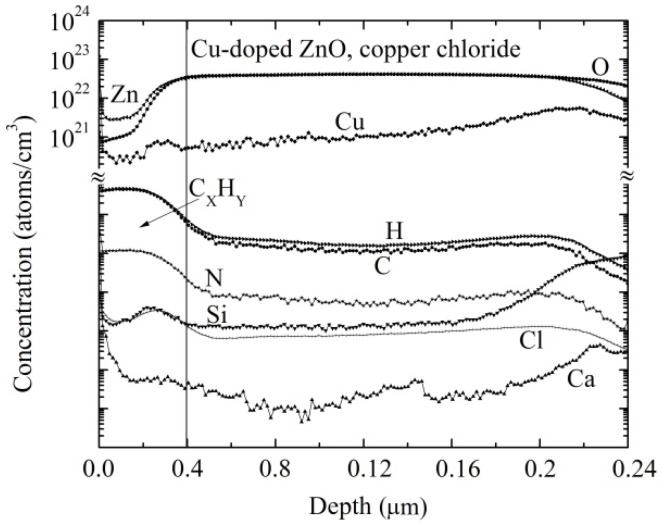
SIMS depth profiles for the Cu-doped ZnO film obtained from six dips in copper chloride and tested for propane sensing.

In Figure 8, a study of the composition (SIMS) for the same Cu-doped ZnO film right after sensing propane at a concentration of 500 ppm and an operating temperature of 300 °C is made. The SIMS depth profile shows the formation of a 40 nm-thick layer of carbon and hydrogen (C*_x_*H*_y_*). We believe this is because the operating pressure never reaches high pressures close to the vapor pressure of the liquid, so it is unlikely to cover the whole Cu-doped ZnO surface, and it is less likely that multilayers are formed. As the surface is porous, the adsorbed gas molecules must have been confined between these pores to a depth of 40 nm. Due to the lack of reference patterns for C, H, Si, N, Cl and Ca atoms, only the depth profiles are shown.

**Figure 9:**
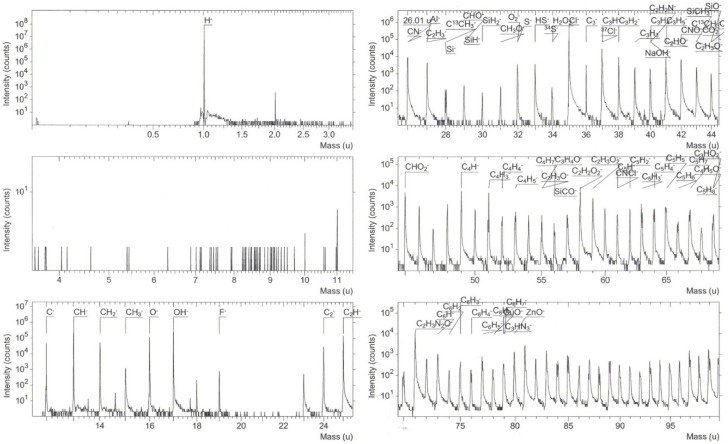
Mass spectrometry for the Cu-doped ZnO film obtained from six dips in copper chloride and tested for propane sensing.

To determine the chemical composition of the Cu-doped ZnO surface, mass spectrometry was performed, and various types of molecules were detected using cesium primary ions for negative secondary ion mass spectrometry, as shown in Figure 9. The results show that for a given element, its corresponding isotopes could also be found; for example, carbon can be found as ^24^C^−^ and ^36^C^−^. A number of molecules that appear to be fragments of the same molecule of propane, C*_x_*H*_y_*, were also found, but some of these fragmented molecules would have reacted with elements, such as O, N and Cl, among others, giving rise to molecules, such as C*_x_*H*_y_*O*_z_*, C*_x_*H*_y_*N*_z_*, C*_x_*H*_y_*N*_z_*O and other heavier molecules still remaining to be specified. It is noticed from Equations 1 and 2 the type of dissociated gas molecule, but from Figure 9, it is understood that multiple chemical reactions (with possible intermediate reactions) can occur, giving rise to fragmented molecules that are associated with oxygen desorbed from the surface of Cu-doped ZnO, such as [CHO]^−^, [CH_3_O]^−^, [H_2_O]^−^, [NaOH]^−^, [C_2_NO]^−^ and [CNO]^−^, among others. Each oxygen atom adsorbed on the Cu-doped ZnO surface releases one or two electrons into the conduction band. These dissociated molecules have an effect on the electrical conductivity of ZnO films. Therefore, the high sensitivity is explained on the basis of the high reactivity of propane on the surface of the Cu-doped ZnO. On the other hand, no copper was observed either as a single molecule or bonded to another atom, which can be due to the configuration of the SIMS system that detects negative secondary ions, *i.e.*, normally the metals are positively ionized.

**Figure 10:**
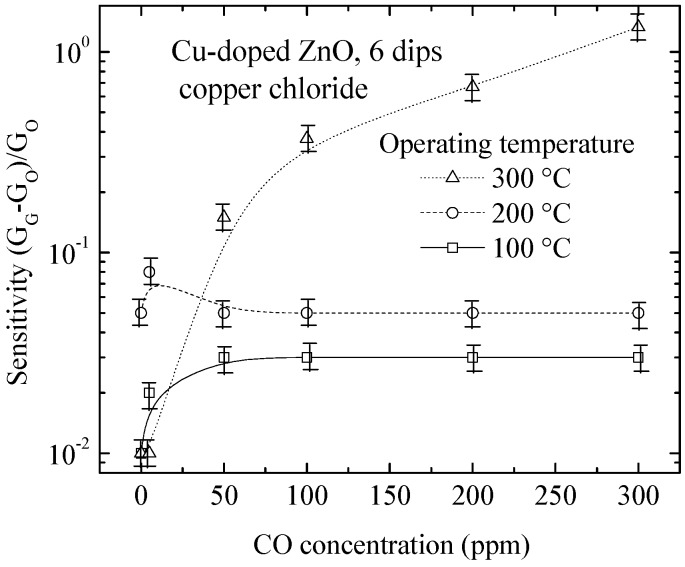
Sensitivity as a function of CO concentration at different operation temperatures for Cu-doped ZnO films prepared from six dips in copper chloride.

To compare the sensing performance of these Cu-doped ZnO films, sensing measurements as a function of the concentration of carbon monoxide (CO) at three operating temperatures for a sample of Cu-doped ZnO obtained from six dips in zinc acetate and using copper chloride were taken and are shown in Figure 10. From this figure, it is observed that the Cu-doped ZnO films sensitivity is less in the presence of the CO gas. This decrement is because the CO molecule has only one carbon-oxygen bond, so it is less complex than the propane molecule, which has eight carbon-hydrogen bonds and two carbon-carbon bonds. Furthermore, larger molecules, like propane, can be fragmented into several parts; they have more chances to bind to the surface-chemisorbed oxygen. Thus, propane has a higher sensitivity than carbon monoxide, as shown in Figure 4, Figure 5 and Figure 10. This deduction is a primary proposal to explain the sensitivity behavior, but other authors have reported different behaviors of sensitivity using propane and carbon monoxide gas on the ZnO surface. Therefore, further studies are necessary to explicate the accurate sensing mechanism with different gases. For example, these results could be complemented with other analytical techniques for the detection of molecules, such as electron spectroscopy for chemical analysis (ESCA), to define more precisely the composition of elements found on the surface (e.g., to corroborate the possibility that oxygen is bonded with copper) and to observe the effect on the electrical conductivity. 

A limiting factor is the measurement setup for CO detection, which allows reaching a gas concentration of 300 ppm. It is observed that the sensitivity does not exceed 1.5, and we believe that following this trend, the sensitivity value does not exceed 2000 for gas concentrations of 500 ppm. This sensitivity value corresponds to undoped ZnO films (refer to Figure 6) [57].

## 4. Conclusions

Cu-doped ZnO films were deposited by sol-gel, using zinc acetate as the zinc salt and two different doping precursors (copper acetate and copper chloride) as the catalyst. The C_3_H_8_ gas sensing properties of Cu-doped ZnO thin films with respect to the operating temperature and gas concentration were analyzed.

Undoped ZnO and Cu-doped ZnO thin films show similar XRD patterns, *i.e.*, the crystal structure of the undoped ZnO films is modified slightly with the introduction of the Cu into the crystal lattice, as these Cu atoms occupy different sites in the atomic lattice of ZnO. From the SIMS measurements, the estimated concentration of copper was found to be 6.2 × 10^20^ and 2.5 × 10^21^ cm^−3^ for ZnO films doped using copper chloride and copper acetate, respectively.

Cu-doped ZnO films prepared from copper acetate and deposited with eight immersions exhibit large sensitivity values (~120) at the operating temperature of 300 °C and a C_3_H_8_ concentration under 300 ppm, whereas at the same operating temperature, but with a C_3_H_8_ concentration over 300 ppm, the maximum sensitivity corresponds to that of Cu-doped ZnO films prepared from copper chloride and deposited with eight immersions. This is because the different doping precursors modify the surface morphology of the film in different ways, which affects the adsorption energy and effective area or contact area of the gas.

X-ray measurements and SIMS analysis were performed on the Cu-doped ZnO post-sensed films, and it was found that for X-ray measurements with increasing operating temperature and a gas concentration of 500 ppm of gas, the crystallinity of the ZnO films worsens, indicating that this adsorption is chemisorption.

From the SIMS measurements, it is observed that a thin layer of apparently 40 nm is formed mainly by the elements of carbon and hydrogen. From mass spectrometry measurements, it was determined that this layer was formed by molecules that might be fragments of the propane molecule, and many of them react with the desorbed oxygen and ultimately affect the electrical conductivity. Therefore, the dominant mechanism of adsorption is the dissociation of gas molecules. Maximum sensitivity values on the order of 4700 and 59,000 were registered for Cu-doped ZnO thin films using copper acetate and copper chloride as catalysts, respectively.
